# Appraising and applying evidence about a diagnostic test during a performance-based assessment

**DOI:** 10.1186/1472-6920-4-20

**Published:** 2004-10-13

**Authors:** George Bergus, Scott Vogelgesang, Janeta Tansey, Ellen Franklin, Ronald Feld

**Affiliations:** 1 Department of Family Medicine, The University of Iowa, Iowa City, IA, 52242, USA; 2 Department of Internal Medicine, The University of Iowa, Iowa City, IA, 52242, USA; 3 Department of Psychiatry, The University of Iowa, Iowa City, IA, 52242, USA; 4 The Office of Student Affairs and Curriculum, Carver College of Medicine, The University of Iowa, Iowa City, IA, 52242, USA; 5 Department of Pathology, The University of Iowa, Iowa City, IA, 52242, USA

## Abstract

**Background:**

The practice of Evidence-based Medicine requires that clinicians assess the validity of published research and then apply the results to patient care. We wanted to assess whether our soon-to-graduate medical students could appraise and apply research about a diagnostic test within a clinical context and to compare our students with peers trained at other institutions.

**Methods:**

4^th ^year medical students who previously had demonstrated competency at probability revision and just starting first-year Internal Medicine residents were used for this research. Following an encounter with a simulated patient, subjects critically appraised a paper about an applicable diagnostic test and revised the patient's pretest probability given the test result.

**Results:**

The medical students and residents demonstrated similar skills at critical appraisal, correctly answering 4.7 and 4.9, respectively, of 6 questions (p = 0.67). Only one out of 28 (3%) medical students and none of the 15 residents were able to correctly complete the probability revision task (p = 1.00).

**Conclusions:**

This study found that most students completing medical school are able to appraise an article about a diagnostic test but few are able to apply the information from the article to a patient. These findings raise questions about the clinical usefulness of the EBM skills possessed by graduating medical students within the area of diagnostic testing.

## Background

Evidence-based medicine (EBM) has been described as the "conscientious, explicit, and judicious use of current best evidence in making decisions about the care of individual patients" [1]. Thus, to practice EBM clinicians need to critically appraise articles in the medical literature and then apply the evidence to specific patients. In the area of diagnostic testing, EBM requires the use of Bayesian inference so that appraised evidence can be used in the evaluation of a specific patient [2].

Nearly all medical schools now provide their students with instruction on EBM [3]. Students at the University of Iowa Carver College of Medicine receive instruction on EBM during required course work during their two preclinical years. Medical students are introduced to EBM in a series of lectures in the first year, including one on the evaluation of diagnostic tests that introduces students to the concept of test characteristics and probability revision. Other lectures focus on critiquing the medical literature. In later semesters, students are asked to complete evidence-based projects and are given further training on the use of Bayes' Theorem. In addition, our students have a required two-week Laboratory Medicine clerkship taken during the third or fourth year. With the aid of a clinically oriented textbook [4], students on this rotation are expected to master the concepts underlying diagnostic testing. To complete this clerkship, students need to demonstrate comprehension of test performance characteristics and probability revision by passing an exam in which they are asked to calculate the sensitivity, specificity, positive predicative value, and negative predictive value for two different testing procedures.

In undertaking this research, we wanted to assess the critical appraisal and probability revision skills of our medical students shortly before graduation within the domain of diagnostic testing. We specifically wanted to evaluate medical students who had previously demonstrated competency at probability revision in a classroom setting. Because EBM is designed to support the delivery of clinical care, transfer of these skills from the classroom to the exam room seemed an appropriate measure of instructional success. We were also interested in comparing the skills of our students nearing graduation to physicians just entering the Internal Medicine residency at our medical center with the intention of comparing the skills of our students to those of students who had trained at other medical schools.

## Methods

### The setting

The data were collected during a performance-based assessment utilizing standardized patients (SPs). During this assessment, subjects had a series of 15 minute encounters with SPs followed by a 10 minute post-encounter activity, thus making each station 25 minutes in length. A 25 minute non-SP based station was integrated into this assessment during which subjects were asked to read and appraise an article about a diagnostic test and apply the information to a preceding SP encounter.

### The subjects

Two different groups of subjects participated in this research. The first group was composed of medical students who were assigned to the Psychiatry clerkship in the late winter and spring of 2003. The second group was composed of all incoming first-year Internal Medicine residents who were in the process of orienting to the residency in June 2003 in preparation for their clinical duties.

### The task

Subjects were asked to critically appraise a research study about a diagnostic test using a worksheet derived from the article about diagnostic tests published in Users' Guides to EBM series [5]. They were asked to assess the validity of the study by identifying the reference standard, whether there was independent and blinded comparison with the reference standard, whether the results of the test being evaluated influenced the decision to perform the reference standard, and whether all subjects underwent the reference standard as well as the test being evaluated. Subjects were also asked to evaluate whether the setting of the study was similar to a community setting in which they would anticipate using the new diagnostic test. Lastly, the subjects were asked to identify the results of the study in terms of the sensitivity and specificity of the test. Thus, in critically appraising the study, subjects were asked to answer a series of 6 questions derived from the published diagnostic test EBM user's guide. After they had assessed the study, the subjects were asked to apply the results of the study by revising the probability of disease given a specified pre-test probability and a test result. To assist with this calculation subjects were provided with calculators.

### The articles

The fourth year medical students, who were participating in their Psychiatry clerkship, were asked to assess an article about a questionnaire to aid in the diagnosis of Major Depression and Panic Disorder [6]. The Internal Medicine residents read an article about a blood test to aid in the diagnosis of congestive heart failure [7]. While the articles focused on different diseases and diagnostic tests, the studies were similar from the critical appraisal perspective. Both articles described research on a new diagnostic test that had been undertaken in carefully selected clinical settings to avoid spectrum bias. Clinical experts who were blinded to the result of the test being evaluated were used as gold standards by both studies. The protocols used by both projects avoided referral bias. Lastly, both articles had been recently published by major medical journals.

### Analysis

For this study, the calculated posttest probability was considered correct if it was ±5% of the correct answer derived from Bayes' Theorem. The performances of the medical students and the Internal Medicine residents were compared using Fisher's Exact Test for dichotomous outcomes and t-test for continuous outcomes. Cronbach's alpha was used to calculate the internal reliability of the 6-item critical appraisal worksheet for each group of subjects. The analyses were undertaken using NCSS 2004 (Kayesville, UT). An alpha of 0.05 was used and all tests were 2-tailed. Approval from the institutional review board was obtained for this project.

## Results

Thirty-eight medical students on the Psychiatry clerkship completed the critical appraisal exercise. Twenty-eight of these students were 4^th ^year medical students who had demonstrated mastery of Bayesian probability revision during a preceding Laboratory Medicine clerkship. The data from these students were analyzed for this report. The other 10 students were dropped from the analysis because they were either M3s or M4s who had not yet completed their Laboratory Medicine clerkship.

Twenty-two first year Internal Medicine residents participated in this performance-based assessment and completed the EBM appraisal task and Bayesian inference exercises during their clinical skills assessment. For this report we used the data from the 15 residents who were recent graduates of US medical schools. The data from the 7 non-US graduates were dropped from the analysis.

Twenty-six of the 28 medical students (93%) correctly identified whether there was an independent, blind comparison of the test with a reference standard (Table 1). Seventeen (61%) correctly identified the reference standard. Eighteen (65%) correctly assessed whether referral bias was present, and twenty-eight (90%) were able to comment on the generalizability of the study. Twenty-five (89%) of the students were able to identify the sensitivity and specificity of the test. The internal reliability of the 6-item critical appraisal questionnaire was 0.59. On average, a medical student correctly answered 78% of the questions related to the appraisal of the article. However, only one of the 28 students (4%) was able to correctly revise the pretest probability.

**Table 1:** Percentage of learners successfully completing tasks relevant to critical appraisal of a diagnostic test journal article

	% Answering Correctly		
Critical Appraisal Guides	Medical Students (n = 28)	IM Residents (n = 15)	P value

Independent, Blinded Comparison With Reference Standard	93%	100%	0.53
Identification of the Reference Standard	61%	53%	0.75
Assessment of Referral Bias	64%	73%	0.74
Completeness of Testing of Subjects	74%	80%	0.72
Generalizability of Results to Typical Practice Settings	89%	93%	1.0
Identification of Sensitivity/Specificity	89%	87%	1.0

All 15 residents (100%) correctly identified whether there was an independent, blind comparison of the test with a reference standard. Eight (53%) correctly identified the reference standard. Eleven (73%) correctly assessed whether referral bias was present, and fourteen (93%) were able to comment on the generalizability of the study. Thirteen (87%) of the Internal Medicine residents were able to identify the sensitivity and specificity of the test for congestive heart failure. The internal reliability of the 6-item appraisal work sheet was 0.66 for this group of subjects. On average, a resident, who had just recently graduated from a US medical school, correctly answered 81% of the questions related to critical appraisal but none of the 15 residents (0%) were able to revise the pretest probability given a test result.

### Comparison of medical students and residents

Overall, the performances of the two groups of subjects were very similar. We did not find any significant differences in their ability to critically appraise an article about a diagnostic test. Medical students, on average, correctly answered 4.7 of the 6 questions related to appraisal while residents correctly answered 4.9 of these questions (p = 0.67). The two groups also showed similar performance on each of the 6 items on the worksheet as shown in Table 1 (p < 0.05 for each) Both groups also showed similar poor performance when asked to revise a pretest probability of disease given the result of a diagnostic test (p = 1.0) using data provided as probabilities.

## Discussion

Over the past decade, EBM has become a major driving force world wide, impacting medical education, policymaking, and research. The teaching of evidence-based medicine has been increasingly integrated into curricula at all levels of medical education as advocated by the Medical School Objectives Program developed by the Association of American Medical Colleges (AAMC) [8]. Like most medical schools, the Carver College of Medicine at the University of Iowa has integrated EBM into its curriculum and our data indicates that students who are soon to graduate can demonstrate proficiency at critically appraising an article about diagnostic testing. But in a simulated clinical encounter, few students are able take the last step of using EBM in diagnosing medical illness. We found almost uniform failure of our students to correctly revise a pretest probability of disease given a test result despite their earlier demonstration of competency with Bayes' Theorem on their Laboratory Medicine clerkship. This finding raises questions about whether our students can fully utilize their EBM training in the clinical setting.

Our finding that incoming residents demonstrate similar levels of skill at critically appraisal and also are unable to revise a pretest probability implies that our medical students' skill deficit is not solely due to a local curricular problem. Because the residents had only just graduated from 12 different US medical schools, this finding suggests that many medical school graduates are able to critically appraise articles on diagnostic testing but few are able to take the next step- that of revising the probability of disease given a test result.

There are few other assessments of the EBM skills of graduating medical students using simulated clinical encounters. In an earlier study, 3^rd ^year medical students demonstrated good performance at critically appraisal [9], a finding which is similar to ours. These results generally replicated classroom-based studies on the success of critical appraisal instruction [10]. To our knowledge, only one other study has investigated whether students are able to integrate critically appraised information about a diagnostic test into clinical decision making. The results of this earlier study also raised concerns of students' abilities to transfer their EBM skills to simulated clinical encounters [11].

Our study has a number of limitations. The first is the very small sample size. However, it is unlikely that a larger sample size would change our conclusion that by the end of medical school students have largely mastered critical appraisal of an article on diagnostic testing but are unable to use this information to revise a patient's probability of disease. A second limitation is that these data were collected at only one medical school. However, as we find the same pattern of competencies in the recently graduated students who are entering our Internal Medicine program it is likely that our findings apply to many other medical schools. A third limitation is that we had our two groups of subjects critically appraise two different articles although they were very similar from this perspective. A final limitation is that it is possible that our subjects would have demonstrated competency in probability revision if we had provided them with a Bayes nomogram or computer spreadsheet. But we wanted to assess whether our students could apply EBM skills to a clinical encounter without any other external supports except for a simple calculator. In the same way, we do not allow our students to take handbooks or other work aids into their 15 minute OSCE encounters with standardize patients.

The poor performance of our students and residents at probability revision is worrisome although previous studies have shown that many clinicians do not master Bayesian inference. Nearly 25 years ago, Casscells documented that few students or faculty at Harvard Medical School were able to correctly complete a probability revision problem [12]. Eddy duplicated this finding in a second group of physicians [13]. However, some cognitive psychologists suggest that humans have most likely always used Bayesian inference in order to survive in our uncertain world. They argue that it is the probability format of the numbers and not the inference task that makes most people fail at the task [14]. A promising line of research suggests that learners show sustained mastery of Bayesian inference using probabilities if they are taught how to first translate probabilities into natural frequencies [15]. Whether this will prove to be the solution deserves study.

## Conclusions

Currently, most of our medical students are able to critically appraise research articles about diagnostic testing but few are able to apply this information at the patient level using Bayesian inference. Because we are able to document the same pattern of skills in entering Internal Medicine residents, we expect this lack of competence with probability revision to be wide spread amongst medical learners. This raises concerns about the clinical utility of the EBM training many students are currently receiving within the domain of diagnostic testing.

## Competing interests

The authors declare that they have no competing interests.

## Authors' contributions

GB conceived of the study. GB, SV, JT, EF participated in study design. GB, EF, RF participated in data collection. GB performed the data analysis. GB, SV, ST, EF, RF participated in drafting the manuscript. All authors read and approved the final manuscript.

## Pre-publication history

The pre-publication history for this paper can be accessed here:



## References

[B1] Sackett DL, Rosenberg WM, Gray JA, Haynes RB, Richardson WS (1996). Evidence based medicine: what it is and what it isn't. Bmj.

[B2] Ashby D, Smith AF (2000). Evidence-based medicine as Bayesian decision-making. Stat Med.

[B3] American Association of Medical Colleges, AAMC Hot Topics in Medical Education.

[B4] Sackett David L. (1991). Clinical epidemiology : a basic science for clinical medicine.

[B5] Jaeschke R, Guyatt G, Sackett DL (1994). Users' guides to the medical literature. III. How to use an article about a diagnostic test. A. Are the results of the study valid? Evidence-Based Medicine Working Group. Jama.

[B6] Spitzer RL, Kroenke K, Williams JB (1999). Validation and utility of a self-report version of PRIME-MD: the PHQ primary care study. Primary Care Evaluation of Mental Disorders. Patient Health Questionnaire. Jama.

[B7] Maisel AS, Krishnaswamy P, Nowak RM, McCord J, Hollander JE, Duc P, Omland T, Storrow AB, Abraham WT, Wu AH, Clopton P, Steg PG, Westheim A, Knudsen CW, Perez A, Kazanegra R, Herrmann HC, McCullough PA (2002). Rapid measurement of B-type natriuretic peptide in the emergency diagnosis of heart failure. N Engl J Med.

[B8] Learning Objectives for Medical Student Education: Guidelines for Medical Schools. http://www.aamc.org/meded/msop/start.htm.

[B9] Davidson RA, Duerson M, Romrell L, Pauly R, Watson RT (2004). Evaluating evidence-based medicine skills during a performance-based examination. Acad Med.

[B10] Norman GR, Shannon SI (1998). Effectiveness of instruction in critical appraisal (evidence-based medicine) skills: a critical appraisal. Cmaj.

[B11] Schwartz A, Hupert J (2003). Medical students' application of published evidence: randomised trial. Bmj.

[B12] Casscells W, Schoenberger A, Graboys TB (1978). Interpretation by physicians of clinical laboratory results. N Engl J Med.

[B13] Eddy DM, Kahneman D, Slovic P and Tversky A (1982). Probabilistic reasoning in clinical medicine: Problems and opportunities. Judgment under uncertainty: Heuristics and biases.

[B14] Gigerenzer G, Goldstein DG (1996). Reasoning the fast and frugal way: models of bounded rationality. Psychol Rev.

[B15] Kurzenhauser S, Hoffrage U (2002). Teaching Bayesian reasoning: an evaluation of a classroom tutorial for medical students. Med Teach.

